# Pituicytoma associated with Cushing’s disease: a case report and literature review

**DOI:** 10.1093/jscr/rjaa104

**Published:** 2020-06-15

**Authors:** Assem S A l rumeh, Mohamed Bafaqeeh, Syed J Allahu khairan, Wafa Al shakweer

**Affiliations:** 1 Pathology and Clinical Laboratory Administration Department, King Fahad Medical City, Riyadh, Saudi Arabia; 2 Pathology and Clinical Laboratory Medicine Administration, Prince Sultan Military Medical City, Riyadh, Saudi Arabia; 3 Department of Neurosurgery, National Neuroscience Institute, King Fahad Medical City, Riyadh, Saudi Arabia; 4 Radiology Department, King Fahad Medical City, Riyadh, Saudi Arabia

**Keywords:** Pituicytoma, Pituitary microadenoma, Cushing's disease

## Abstract

Pituicytoma is a rare tumor that has been recently recognized and described, where only few reported cases of pituicytoma associated with Cushing’s disease. We describe a case of a 47 years old female with a history of high cortisol levels and a diagnosis of Cushing’s disease was made. Brain magnetic resonance imaging showed lesion in pituitary gland compatible with microadenoma and tumor resection was carried out. The histopathological findings were of a pituicytoma with positive thyroid transcription factor-1 immunostain.

## INTRODUCTION

Pituicytoma is an extremely rare neoplasm of the sellar and suprasellar region that arising from the neurohypophysis or infundibulum. This tumor was described for the first time in the literature in 1955, and only 78 cases of pituicytoma have been reported [1]. In 2007, pituicytoma was described as a low-grade tumor (Grade I) according to the World Health Organization classification of central nervous system tumors [2]. The tumor can often mimic pituitary adenoma and also associated with Cushing’s disease [3, 4]. Other clinical features include compression of the optic chiasm, infundibulum and/or pituitary gland causing visual disturbance; headache; and features of hypopituitarism such as fatigue, amenorrhea, decreased libido and mildly elevated serum prolactin [2, 5]. On magnetic resonance imaging (MRI), the tumor is typically solid and circumscribed mass lesion of the sellar and suprasellar spaces. Most often, it is isointense to gray matter on T1-weighted images, hyperintense on T2-weighted images and uniformly contrast enhancing. Clinical manifestations and radiological finding are non-specific; diagnosis is typically made on the basis of histopathological results and immunohistochemical (IHC) examinations [1]. Here, we present the clinical presentations, pathological features and treatment outcome in addition to review of the literatures.

**Figure 1:**
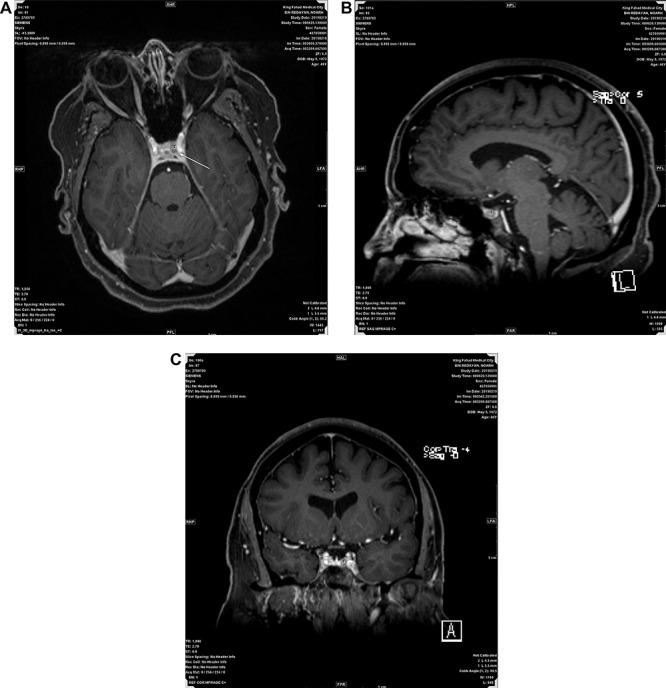
(**A**, **B** and **C**) Represent post contrast Axial, Sagital and Coronal MRI images respectively, showing a small non enhancing nodule in left side of adenohypophysis measuring 5.5 mm suggestive of microadenoma.

## CASE REPORT

**Figure 2:**
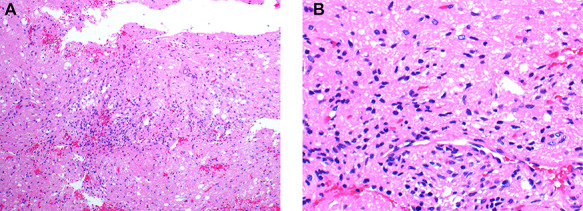
(**A&B**) Histopathological examination of the pituicytoma specimen shows elongate, bipolar spindle cells arranged in a fascicular and storiform patterns with no significant nuclear pleomorphism,mitotic activity and necrosis.

**Figure 3:**
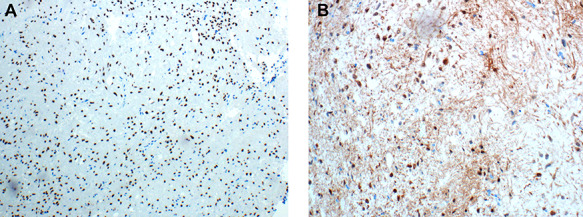
(**A&B**) Immunohistochemical examination of the pituicytoma specimen shows strong and diffuse nuclear staining for TTF-1 (Figure 3A), and rare cells shows positive staining for S100 (Figure 3B).

A 47 years old female known case of type 2 diabetes mellitus and Hypertension presented with muscle weakness and gain weight. On physical examination, the patient had large, rounded face (moon face), increased fat in her neck and shoulder areas (buffalo hump) and with thinner arms and legs (central obesity). Her laboratory investigations showed high cortisol level (489 nmol/L) with normal Adrenocorticotropic hormone (ACTH) plasma level (11.1 pmol/L). MRI scans showed a 5 mm solid mass in left side pituitary gland. Figure 1A–C representing post contrast axial, sagittal and coronal images, respectively, showed a small non-enhancing nodule in left side of adenohypophysis of the pituitary gland measuring ~5.5 mm. Imaging features suggested microadenoma. The patient underwent transsphenoidal pituitary tumor resection. The histopathological findings were of H&E stained sections showing multiple fragments of tumor tissue exhibiting elongated and bipolar spindle cells arranged in a fascicular and storiform patterns with adjacent unremarkable pituitary gland. The individual tumor cells contained slightly fibrillary eosinophilic cytoplasm with oval nuclei (Fig. 2A and B). A panel of IHC studies was done, which revealed that the spindle neoplastic cells are positive for thyroid transcription factor-1 (TTF-1) (diffuse nuclear staining), S100 and glial fibrillary acidic protein (GFAP) (rare cells) (Fig. 3A and B). These findings are consistent with a pituicytoma.

## DISCUSSION

Pituicytoma is a rare, low-grade glial neoplasm originating in the neurohypophysis or infundibulum. The tumor can often mimic pituitary adenoma and also associated with Cushing’s disease. Like spindle cell oncocytomas and granular cell tumors of the sellar region, pituicytomas show nuclear expression of TTF-1, suggesting that these three tumors may constitute a spectrum of a single nosological entity [2]. A similar case to our case reported by Xiaopeng Guo in Department of Neurosurgery, Peking Union Medical College Hospital, Chinese Academy of Medical Sciences, a 46-year-old Chinese female who developed signs and symptoms of Cushing’s syndrome, including supraclavicular and dorsocervical fat pads, a moon-shaped face with thin extremities, severe fatigue, weakness and memory dysfunction for 2 years. MRI revealed an abnormally enlarged parenchymal lesion in the sellar region (especially in the left inferior pituitary gland), measuring 15 mm. Histopathological examination showed tumor composed of bipolar spindle-shaped cells. No significant nuclear polymorphism and mitosis. The IHC profile showed diffuse positivity for GFAP, nuclear factor and S100. IHC stain for ACTH hormone showed strongly positive, at this point, a pituicytoma coexisting with ACTH-secreting pituitary hyperplasia was finally diagnosed [3]. Another two causes of pituicytoma were published in Journal Oncology Letters. First case was a 47-year-old female with history of headaches, menstrual disorder and slightly elevated prolactin level (45 ng/ml). MRI scans showed a 16 × 13 × 10 mm solid mass. Second case was a 51-year-old female presented with 1 year of visual complaints, 6 months of diabetes insipidus and 2 months of headaches. Visual field testing showed left temporal hemianopsia with elevated prolactin level (188 ng/ml). The histopathology and IHC studies in both cases showed similar findings seen in our case [5].

Edward B. Lee described three cases of pituicytomas, four cases of granular cell tumors and eight cases of spindle cell oncocytomas. Strong expression of thyroid transcription factor-1 immune stain was obtained in all of the 15 cases [6].

In our case, the tumor was so tiny ~0.5 cm that it could be missed as a fibrous stroma or meningeal tissue but being aware of this entity and having TTF-1 positivity helped us to reach to the definitive diagnosis.

In conclusion, pituicytoma should be included in the differential diagnosis of spindle cell neoplasm of the sella turcica, meningioma, schwannoma, solitary fibrous tumor, hemangiopericytoma, glioma, tanycytic ependymoma and Epstein-Barr virus (EBV)-associated smooth muscle tumor. Pituicytoma is a unique tumor in terms of intimate relation to the pituitary gland, the glial nature and uniform expression of TTF-1 antigen.

## Conflict of interest statement

There are no conflicts of interest.

## References

[1] Scothorne CM A glioma of the posterior lobe of the pituitary gland. J Pathol Bacteriol 1955;69:109–12. 13243176 10.1002/path.1700690115

[2] Brat DJ, Scheithauer BW, Fuller GN, Tihan T Newly codified glial neoplasms of the 2007 WHO classification of tumours of the central nervous system: angiocentric glioma, pilomyxiod astrocytoma and pituicytoma. Brain Pathol 2007;17:319–24. 17598825 10.1111/j.1750-3639.2007.00082.x PMC8095654

[3] Guo X, Fu H, Kong X, Gao L, Wang W, Ma W, et al. Pituicytoma coexisting with corticotroph hyperplasia. Medicine (Baltimore) 2016;95:e3062 http://www.ncbi.nlm.nih.gov/pmc/articles/PMC4998918. 26962837 10.1097/MD.0000000000003062 PMC4998918

[4] Schmalisch K, Schittenhelm J, Ebner FH, Beuschlein F, Honegger J, Beschorner R Pituicytoma in a patient with Cushing's disease: case report and review of the literature. Pituitary 2012;15:S10–6. 20945102 10.1007/s11102-010-0262-3

[5] Mao Z, Xiao W, Wang H, Li Z, Huang Q, He D, et al. Pituicytoma: report of two cases. Oncol Lett 2011;2:37–41. www.ncbi.nlm.nih.gov/pmc/articles/PMC3412475. 22870125 10.3892/ol.2010.209 PMC3412475

[6] Lee EB, Tihan T, Scheithauer BW, Zhang PJ, Gonatas NK Thyroid transcription factor 1 expression in sellar tumors: a histogenetic marker? J Neuropathol Exp Neurol 2009;68:482–88. 10.1097/NEN.0b013e3181a13fca 19525896

